# Chikungunya in Mozambique: A Forgotten History

**DOI:** 10.1371/journal.pntd.0005001

**Published:** 2016-11-17

**Authors:** Eduardo S. Gudo, James F. P. Black, Julie L. Cliff

**Affiliations:** 1 National Institute of Health, Ministry of Health, Maputo, Mozambique; 2 Nossal Institute for Global Health, Melbourne School of Population and Global Health, University of Melbourne, Melbourne, Australia; 3 Community Health Department, Faculty of Medicine, Eduardo Mondlane University, Maputo, Mozambique; University of Texas Medical Branch, UNITED STATES

## Introduction

The control of any infectious epidemic- or outbreak-prone disease demands a thorough understanding of geographic distribution in both endemic and epidemic settings. This may require a careful study not only of recent reports but also of activity far back into the historical record. For chikungunya, the historical record provides evidence of the presence of the virus in Mozambique. Recently, serological evidence of chikungunya, based on seroconversion and a 4-fold rise in titer of IgG antibodies in acute febrile patients in southern Mozambique, has been found [1]. No epidemic of chikungunya has been reported in Mozambique since 1952–1953.

This paper revisits earlier published and unpublished work on chikungunya in Mozambique. In addition, we discuss the name of the disease, which may provide a clue to its presence.

With the reemergence of chikungunya in 2000 after several decades of absence, new attention has been drawn to the virus. Between 2005 and 2007, large epidemics were reported in the Indian Ocean islands of the Comoros, Seychelles, Mauritius, and Reunion [2]. The Comoros lie just across the Mozambican channel from northern Mozambique, and interchange is frequent.

## History of Chikungunya in Mozambique

Both parts of the published description of the first recorded chikungunya epidemic in 1952–1953 in Tanzania (then Tanganyika) include reports of cases in Mozambique (then known also as Portuguese East Africa). Robinson [3], describing the clinical details, cites a report from Dr. Carlos Santos de Reis of large numbers of cases in the coastal town of Moçimboa da Praia. In the second part, Lumsden [4], describing the epidemiology, includes information from Dr. Fritz Bauer, who had visited northern Mozambique:

“Migrant workers coming to Newala from Portuguese East Africa reported before the end of February that a dengue-like disease was rife on the Mawia Plateau (N.J. SINCLAIR, personal communication). Dr. Fritz BAUER … informs me that the disease began in the coastal areas in March, 1953, increased in April and ended, following an insecticide campaign, in May. The towns of Quionga, Palma and Mocímboa da Praia were especially affected; more than 60% of the population suffered attack. There were no cases south of Mocímboa da Praia. The outbreak described by Dr. BAUER in the coastal areas was after the main epidemic on the Makonde Plateau and, almost certainly after the Mawia Plateau outbreak also. If the disease was the same, then there is a sharp distinction between Tanganyika and Portuguese East Africa in that in the former territory the coastal districts—although some dengue-like cases were reported—never became seriously involved.”

The two plateaus lie on either side of the Mozambique–Tanzania border, separated by the Rovuma River. Mawia is a pejorative name used for Mozambican Makonde in older Tanzanian literature [5,6].

**Fig 1:**
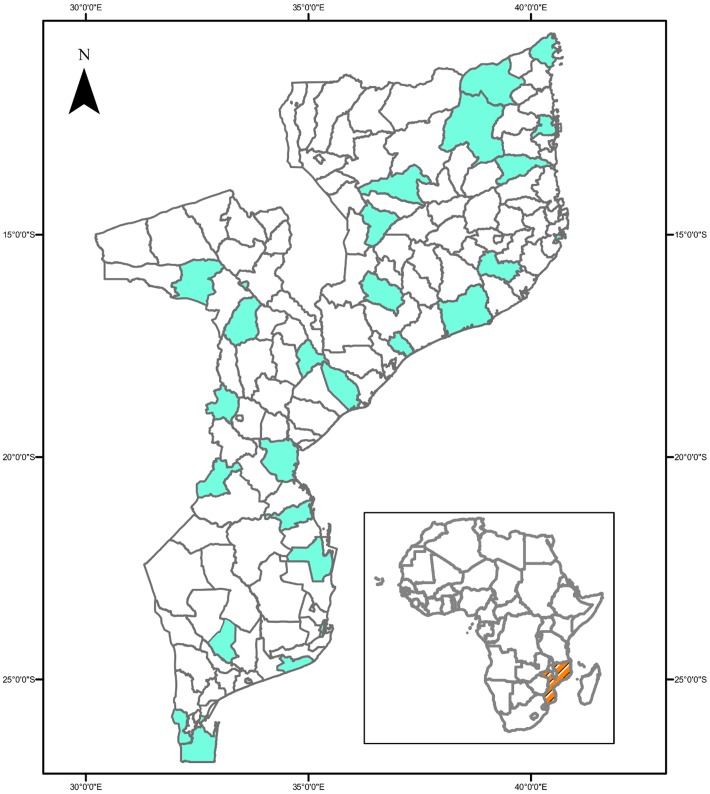
Mozambique, with shaded areas representing districts surveyed (all with positive results) for chikungunya antibodies in 1957. Source of data: Kokernot et al. [7] *Image Credit*: Américo Feriano José.

Since the first reported epidemic, serosurveys in Mozambique have indicated the presence of chikungunya. In 1957, Kokernot et al. [7] carried out a survey using serum neutralization tests in 29 widely dispersed sites. Fig 1 shows the districts surveyed, and Table 1 shows the principal results, together with results from subsequent serosurveys. The serosurvey in 1957 found antibodies against chikungunya in all sites, with an overall prevalence of 21.9% (191/871): 4.0% (16/404) of children and 37.5% (175/467) of adults. The prevalence was higher north of the Zambezi River: 29.0% (114/393) compared to 16.1% (77/478) south of the river. Children and adults south of the river showed prevalences of 2.2% and 28.5%, respectively, and north of the river, 6.1% and 48.1%, respectively. In regions such as Mueda, there was serological evidence of recent viral activity and possible endemicity. Mueda is located on the Mozambican Makonde plateau, where cases were reported in the first epidemic. They concluded that chikungunya had spread through Mozambique in an epidemic wave (Fig 1).

**Table 1:** Chikungunya serosurveys in Mozambique, 1957–1987.

Year	Location	No. of positives/total (%)	Reference
**1957**	South of Zambezi River	77/478 (16.1%)	[7]
	North of Zambezi River	114/393 (29.0%)	
	Total	191/871 (21.9%)	
**1971–1973**	Zambezi River region	Exact number not available	[8]
		200–400 tested each year	
		65%–81% positive	
**1987**	Capital city, provincial capitals		[9]
	South	8/121 (6.6%)	
	Central	5/39 (12.8%)	
	North	11/39 (28.2%)	
	Total	24/199 (12.1%)	

Chapters in major textbooks published in 1975 [10] and 1989 [11] refer to the results of this published survey and therefore to the presence of chikungunya in Mozambique.

In a 1975 memoir, McIntosh [12] stated that chikungunya has a tropical distribution in Southern Africa. An accompanying map, dated 1968, shows a distribution based on antibody surveys in man and wild primates in Botswana, Mozambique, South Africa, South West Africa (now Namibia), and Rhodesia (now Zimbabwe).

In 1977, de Moor et al. [8] noted that “all the epidemics of Chikungunya that have been confirmed by virus isolations in the south-eastern part of the continent (including the first…) have occurred in close proximity to the coastal plain of Mozambique.” The map in their publication shows three additional confirmed epidemic sites close to the Mozambican border and ten widely dispersed sites in Mozambique where more than 50% of the human population had antibodies against chikungunya. They considered it likely that the main focus lay in Mozambique and that epidemics in South Africa, Tanzania, and Rhodesia represented relatively isolated incidents of the spread of disease away from this focus. They therefore investigated the zoology and dynamics of chikungunya transmission in Mopeia, which is on the Zambezi River 100 km from the coast in central Mozambique. Examination of the data available from the previous survey in 1957 [7] showed that 10 of 15 adults and none of 13 children in Mopeia had chikungunya antibodies. The later studies, performed in 1971, 1972, and 1973, included collection of human sera for analysis of chikungunya antibodies at Mopeia and other sites on the Zambezi River near the coast. Sera were collected from between 200 and 400 individuals (the great majority children) each year. Immunity to chikungunya virus was found in between 65% and 81%, with high titers of antibodies throughout (Table 1). They do not state whether they performed neutralization tests, and this may be a limitation of their findings due to the presence of other alphaviruses in the area. They found similar high titers in captured monkeys and baboons. The age of one of the immune baboons indicated that virus activity had taken place in the 18 months prior to its capture in September 1972.

They concluded that the probable explanation for these findings is that chikungunya is endemic in the human and wild primate populations of the Zambezi delta region. Although there was a chance that both sets of surveys had followed immediately after a chikungunya epidemic, this was unlikely. In 1971–1973, they interviewed school teachers, doctors at local hospitals, and personnel at missions and clinics. None reported a recent epidemic. Doctors and medical attendants were, however, familiar with a common illness, occurring sporadically, which resembled chikungunya. Locally called “break-bone disease,” the disease was characterized by severe joint pains and fever.

Their explanation for the findings was that chikungunya was probably endemic and enzootic in humans and wild primates of the central and northern Mozambique coastal plain.

Later, in 1987, serum samples collected from adults for HIV surveillance in the capital city, Maputo, and nine provincial capitals were screened at the National Institute for Virology in South Africa for arboviruses, first by hemagglutination and hemagglutination-inhibition. Reactive sera were further tested by IgG and IgM direct and indirect ELISAs. Table 1 shows that results for chikungunya were 6.6% (8/121), 12.8% (5/39), and 28.2% (11/39) positive in the south, center, and north, respectively [9]. We have no results for neutralization tests, which limits the findings of this survey.

## Chikungunya or Chingwingwinda?

The authors of the first publications gave the name chikungunya, used by the local Tanzanian Makonde people to describe the disease [3,4]. It meant “that which bends up” and was derived from the root verb *kungunyala—*to dry up or become contorted. Chikungunya is often referred to as the Makonde name for the disease and is sometimes erroneously called a Swahili word [2]. Chikungunya is, however, not used by Mozambican Makonde to describe the classic symptoms. In the early 1990s, a Makonde student corrected one of the authors (JLC) when she stated in a lecture that chikungunya was a Mozambican Makonde word. The Makonde language is composed of many different dialects, and the Mozambican dialects are different from those in Tanzania, where the first epidemic was described. Subsequently, another author (JFPB) interviewed Makonde health workers in the northern Mozambican coastal town of Moçímboa da Praia (a site of the first chikungunya epidemic). None associated the word chikungunya with the disease. However, many recognised the disease from a description of the clinical features, and they were unanimous that the correct name is chingwingwinda (pronounced shin-gwin-GWIN-dah). The word was derived from gwingwindar, meaning to “bend up,” and described the characteristic joint flexion. Several described the local remedy: boiled cassava leaves rubbed into the affected joints. In 2014, Makonde health workers interviewed by JLC recognized the name chingwingwinda and were able to describe the classic symptoms.

The continued existence of a specific word for this disease in Mozambican Makonde suggests that the disease has been known on the Mozambican Makonde plateau for some time.

## Conclusions

Although we are aware of no reports of virus isolation from Mozambique, the combined evidence of reported cases in Mozambique during the first chikungunya outbreak in 1952–1953 and results from a serosurvey with neutralization testing in 1957 showing widespread positivity strongly suggest that chikungunya was present in Mozambique. Later studies in the 1970s and 1980s, although limited, suggest a continuing presence of chikungunya.
